# Horse Hoof Trimmings as an Untapped Resource for Sustainable Keratin Utilization

**DOI:** 10.1002/open.202500575

**Published:** 2026-04-14

**Authors:** Esther Trigueros, Sara Mattiello, Lisa Rita Magnaghi, Carlo Santulli, Raffaela Biesuz

**Affiliations:** ^1^ Dipartimento di Chimica Università degli Studi di Pavia Pavia Italy; ^2^ School of Science and Technology Università di Camerino Camerino Italy; ^3^ INSTM Unità di Ricerca di Pavia Firenze Italy

**Keywords:** bio‐based materials, circular chemistry, green extraction, horse hoof trimmings, keratin recovery, sustainable biopolymers, waste valorization

## Abstract

Keratin, a structural protein with outstanding mechanical and biochemical properties, is abundant in animal‐derived wastes such as feathers, wool, and hooves. However, these keratin‐rich materials are still largely incinerated or landfilled, leading to environmental burdens and resource loss. This review introduces horse hoof trimmings as an unexploited, renewable, and cruelty‐free keratin source generated through routine equine care. Unlike slaughter‐derived materials, hoof trimmings provide a traceable and high‐quality biopolymer feedstock with the potential for sustainable valorization. We summarize current knowledge on their chemical composition, structure, and physicochemical properties, highlighting correlations with nutrition, environment, and management practices. Furthermore, we critically assess green extraction methods and upcycling strategies for keratin recovery, identifying challenges and opportunities for scaling toward industrial applications. By focusing on this overlooked waste stream, this review aims to stimulate innovation in sustainable materials chemistry, biopolymer engineering, and circular resource management, advancing the principles of green and circular chemistry.

## Graphical abstract

This review outlines horse hooves as an overlooked, sustainable source of cruelty‐free keratin. It synthesizes current insights into their structure, composition, and properties and highlights the main extraction methods, emphasizing their sustainability and the material's potential applications.

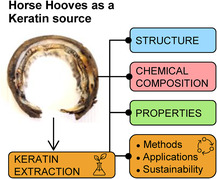

**Abbreviations**
CAGR: Compound annual growth rate
EDTA: Ethylenediaminetetraacetic acid
GS: Greenness Score
IFs: Intermediate filaments
SDS: Sodium dodecyl sulfate

## 1 Introduction

Keratin is the most abundant structural protein in epithelial cells and, following collagen, the second most prevalent biopolymer in humans and animals [1, 2]. Its functions in living organisms include waterproofing, temperature regulation, tissue cohesion and structuring, internal tissue protection, wound healing, and the elimination of toxins and waste, among others, which also suggest biomedical applications [3]. The aforementioned characteristics derive from its very structure, which is based on polypeptide chains stabilized by hydrogen bonds, ionic bonds and disulfide bonds, and hydrophobic interactions. The high level of cross‐linking among different chains can also provide it with remarkable thermal stability [4] and mechanical strength, which decline in various senses according to the microstructure, including flexural, compression, and shear strength and toughness [5].

Keratin is rich in sulfur‐containing amino acids, particularly cysteine (7–13%), which is also present at higher levels compared to other fibrous proteins such as elastin, collagen, and myofibrillar proteins [6]. These sulfur‐rich amino acids form inter‐ and intramolecular disulfide bonds, further enhancing keratin stability. Based on sulfur content, keratin can be categorized as hard or soft, while *α*‐keratin, *β*‐keratin, and the nonstructural *γ*‐keratin are differentiated by their composition and structure [7].

Animal nonedible tissues represent the primary source of keratin. Mammalian sources such as wool, hair, nails, hooves, horns, and claws are significant producers of *α*‐keratin. In contrast, feathers, quills, beaks of birds, and scales from fish and reptiles yield *β*‐keratin [8]. These materials are by‐products of slaughtering and butchering operations in the meat industry, whose production is continuously increasing [9]. For instance, in 2020, livestock‐derived keratin production reached 11.82 million tons, primarily sourced from chicken feathers and wool, which contain approximately 95% and 90% keratin, respectively [10]. Despite their quality and unique properties, keratinous by‐products are often considered wastes and currently need to be treated in specialized sites by incineration or landfilling according to EC Regulation 1069/2009 [11], leading to the emission of greenhouse gases (CO, CO_2_, SO_x_, and NO_x_), volatile organic compounds, ammonia, and nitrogen‐based species that contaminate land, air, and water. Such disposal practices increase environmental hazards, compromise public health due to infection risks, and discard valuable protein resources [12].

To address this issue, it is essential to adopt environmentally sustainable strategies for keratin waste management. Efficient processing and valorization of keratinous materials do not only reduce environmental pollution and carbon emissions but also enhance the sustainability of the meat production system by transforming waste into valuable resources. More recently, end‐of‐waste considerations did suggest the regeneration of this refuse, but few real case pilots can be mentioned: an interesting but niche example is represented by the wet spinning of a keratin solution from discarded post‐service wool [13].

The remarkable mechanical and biological properties of keratin, along with its thermal stability, excellent biodegradability [14] and biocompatibility, and cellular adhesion capabilities, make it an ideal raw material for diverse applications [15]. These include the development of bio‐based materials, functional foods, pharmaceuticals, agricultural products, textiles, and more [16]. The market demand for biopolymers and bioplastics is projected to grow by 24.2% between 2024 and 2029. Keratin can contribute to this demand, thereby reducing the environmental impact of petroleum‐based plastics by the fabrication of keratin‐based biopolymers, particularly using chicken feathers obtained gels [17]. Due to its excellent biocompatibility, keratin‐based materials are widely used in wound healing treatments, antibacterial formulations, and drug and bioactive substances delivery systems, normally in the form of nanoparticles gels [18]. More in general, keratin serves as a low‐cost, nontoxic raw material for developing bio‐based hydrogels, films, fibers, scaffolds, and nanoparticles used in regenerative medicine, drug delivery, and tissue engineering [19]. In cosmetics, keratin‐based materials, such as hydrolysate, enhance the moisture, elasticity, and thermal properties of hair and skin [2, 20].

Beyond the above fields, keratin is employed in wastewater treatment as a biosorbent thanks to its functional groups (amino, carboxyl, hydroxyl, and sulfhydryl), which enable the removal of heavy metals, organic pollutants, and toxic substances from water. Additionally, keratin serves as a biofertilizer in sustainable agriculture, providing carbon, nitrogen, sulfur, and micronutrients that enhance soil quality and plant growth [21]. It is also used as a nutrient‐rich additive for animal feed and human dietary supplements, with high cysteine content. However, the potential of keratin is enormous and only limitedly explored. Other applications in the material field include leather and textile processing as treatment/tanning agent [22], the development of fibers for composite materials, where using various sources of keratin (e.g., chicken feathers, or waste wool fibers) provides distinctly different properties [23], or for fuel cells [24] and electronic devices [25], and the production of flame retardants for thermosetting resins, such as epoxy [26].

Despite its exceptional properties and wide‐range prospective use, keratin remains underutilized due to limited research on industrial‐scale applications, poorly designed extraction processes, and a lack of awareness regarding its advantages over plant‐derived proteins. It is worth mentioning that the protein market has been estimated to grow at a compound annual growth rate (CAGR) of 10.5% (2021–2028). Simultaneously, the keratin market, valued at 1.4 billion USD in 2021, exhibits an estimated CAGR of 6.2% between 2022 and 2030, driven by population growth and industrialization [27]. This rising demand highlights the need for alternative protein sources.

This review aims to comprehensively evaluate animal‐derived keratin with a focus on sources derived from animal waste such as horse hooves, along with its composition, properties, and potential applications to address global protein demand and promote and support the development of sustainable biopolymers and more generally of biomaterials as an alternative, in view of a circular economy approach. More specifically, the potential use of one of the most neglected sources of keratin, i.e., waste deriving from the trimming process for the shoeing of horse hoofs, is examined. The amount of refuse generated, though obviously not comparable with other sources, such as sheep wool and chicken feathers, bears on the other hand the advantage of being prevalently concentrated in reasonably large breeding establishments, therefore easily traceable. However, the knowledge on the structure and possibilities of this secondary raw material is still limited; in parallel with what has been done so far on other types of keratins, dedicated attention needs to be applied to the topic, especially as a suggestive starting point for future exploration on the potential of this filler.

## 2 Methodology

For this review, an extensive search was carried out across the Scopus, PubMed, and Web of Science databases, including articles, review papers, and books from all time periods, though with a preference for literature issued post‐2000, restricted to English‐language publications. The search utilized keywords such as “horse hoof,” “horse hooves,” “cattle hooves,” “equine wall,” “keratin,” “keratinous materials,” “mineral composition,” and “amino acid content.” Following an initial screening, studies were selected based on their relevance to this review. More preference for hard and particulate structures (such as nails) has been given over fibrous keratin structures for their larger resemblance to the target of this work.

The review has been divided into six main sections dedicated to general description of horse hoof trimming waste (Section 3), hints to their morphology and functions (Section 4), chemical composition (Section 5), properties (Section 6), and, lastly, keratin extraction (Section 7) and upcycling potential (Section 8).

## 3 Horse Hooves Trimming Wastes: Unveiling an Alternative Keratin Source

It is fair to say that, as an animal‐derived source of keratin, hooves have been so far overlooked in literature, though the number of cattle hooves generated by the food sector industry is comparable to other animal by‐products [28]. In parallel with cattle hooves wastes from slaughterhouses activities, which means entire hooves are collected after the death of animals meant for human consumption, the scientific community would be supposed also to investigate into the use of horses’ hooves waste derived from shoeing and trimming operations, as sustainable, traceable, high‐quality, and cruelty‐free keratin sources, an exigency that is gradually more valued through the society and influences also the market [29]. As a matter of fact, horse hoof trimmings present several meaningful differences from established feedstocks (e.g., feathers and wool) that can be rapidly summarized in the following key points.

• Cruelty free: trimming or shoeing must be conducted at least once a month for horses’ health and well‐being, both in the case of sport horses involved in various riding disciplines and for those employed in working or reproduction activities [30]. Therefore, these wastes are generated without implying any cruelty toward animals but even aiming at their well‐being.
• Quality and purity: comparing horse hooves to cattle ones, higher quality materials derive from the first waste streams. On one hand, working or sport horses’ basal nutrition, dietary supplements, balanced workout, and proper housing are finely optimized to preserve hooves quality to guarantee the horses’ performance [31, 32, 33, 34, 35]. On the other hand, breeders focus their attention on cattle hooves with the sole aim of avoiding premature death from hoof pathologies, thus resulting in a miscellaneous and low‐quality waste material generated from slaughter‐derived sources [36, 37, 38, 39]. This assumption is confirmed by literature data comparing different materials’ purity: the exceptionally high protein content reported for horse hooves (98.24 to 98.86% on a dry basis) represents the upper end of reported keratin content for animal by‐products, considering around 90% for wool and 95% for feathers [10].
• Traceability and variability: shoeing or trimming activity singularly performed on each horse by specialized farriers and thus allowing for knowledge of the geographical provenience of the waste and any other required detail. Lastly, the wide variety of horse management practices developed over the centuries resulted in an extremely high variability in hoof‐derived material composition and properties [40]. If properly sampled, characterized and elaborated by multivariate approaches, as already presented in investigations on these materials’ composition [41, 42, 43, 44], such variability could offer various benefits and opportunities in the usage of horse hooves waste materials.
• Availability and logistics: since no data are available on trimming waste volume generated worldwide, we estimated a yearly production of around 106 000 tons, considering the 53 million sport horses trimmed or shod 10 times per year and an estimated conservative value of 200 g of hoof trimming waste per event. This latter value is derived from preliminary experimental data from our ongoing research activities and is used in the absence of peer‐reviewed data to establish an initial order of magnitude for this untapped resource. A similar amount is undoubtedly negligible if compared to the yearly production of keratinous animal by‐products, 12 million tons according to Chen and coauthors [10], yet it presents a key logistical advantage: the refuse generated is prevalently concentrated in reasonably large breeding establishments or stables, making the material easily traceable and the collection more efficient for industrial‐scale valorization, compared to widely dispersed feedstocks.
• Structural difference: keratin from horse hooves is classified as a hard, particulate *α*‐keratin, whose unique hierarchical, laminated structure and superior mechanical properties do distinguish it from the fibrous *α*‐keratin of wool or the *β*‐keratin of feathers, opening distinct pathways for bio‐inspired materials and composite development [45].

Before moving to the next section, a clarification on trimming waste volume estimation must be made. It is important to note that this figure, 106 000 tons/year, represents a best case, preliminary estimation and is subject to significant uncertainty. Variables such as the actual ratio of sport/working horses being trimmed (vs. unshod horses), variations in trimming frequency across geographies, and the precise amount of waste generated per event (which depends on hoof condition and farrier technique) introduce a high degree of variability. However, we consider this estimation crucial for framing the resource potential of horse hoof trimmings, and it clearly indicates that this material constitutes a non‐negligible, concentrated feedstock that merits dedicated, quantitative studies for its accurate assessment.

## 4 Structure and Its Functional Impact on Valorization

The horse's hoof is a highly complex biological structure, which has historically been studied to elucidate the biochemical principles behind such a resistant, stiff, and tough material [46]. More recently, researchers have been turning to this material in search of ideas for novel bio‐inspired materials showing similar properties [39]. From a material science perspective, the hoof wall is primarily composed of epidermal cells that synthesize and deposit different types of hard *α*‐keratins [47]. These proteins are organized in a highly compact, hierarchical, and geometrical structure. This design is built upon the extensive molecular cross‐linking (primarily disulfide bonds) that occurs between keratin fibers [48], creating a stable composite of long, thin fibers embedded in a surrounding matrix. The resulting structural tenacity of the hard *α*‐keratin in hoof trimmings has a direct functional consequence on its industrial valorization:

• Extraction constraint: The high density of disulfide bonds and the compact, crystalline nature of the *α*‐keratin necessitate the use of harsh chemical treatments (e.g., strong reducing/oxidizing agents or high temperatures) to achieve full solubilization. This challenge dictates the focus on optimizing green extraction methods to maintain yield while mitigating environmental impact.
• Upcycling advantage: The material is generated as a rigid, fine particulate waste, distinct from the fibrous structures of wool or feathers. This intrinsic morphology could make the resulting keratin powder uniquely suitable as a high modulus reinforcing agent in composite materials, directly transferring the inherent mechanical strength of the native structure to new products.

## 5 Horse Hooves Chemical Composition

### 5.1 Protein Content and Major Amino Acids

Horse hooves have a largely prevalent keratin content, so that the remaining components can be deemed to be in traces: for example, Tocci et al. [40] reported that horse hooves contain a protein content ranging from 98.24 to 98.86% on a dry basis, with no significant variations across different horse breeds. More in detail, the main component of hooves is hard *α*‐keratin, organized in crystalline intermediate filaments (IFs) embedded in an amorphous matrix forming a short‐fiber reinforced biopolymer [49]. In *α*‐keratin, the amino acids form polypeptide chains constituted by a right‐handed *α*‐helix secondary protein structure through the use of hydrogen bonds, of approximately 45 nm length. Two of these polypeptide chains bind to each other to form a coiled‐coil dimer (45 nm length, 2 nm diameter), which constitutes the molecular unit of *α*‐keratin. Then, two dimers bond to form a tetramer, which is in turn bonded to others through disulfide bonds between cysteine residues to form protofilaments. Two protofilaments form a protofibril, and four protofibrils create the crystalline IF, embedded in an amorphous keratin matrix [50]. These IFs within the amorphous matrix are interconnected through peptide and disulfide bonds, assembling into macrofibrils (400–500 nm diameter) and fibers (6 µm). The orientation, alignment, and volume fraction of the IFs, as well as the matrix characteristics, influence the mechanical properties of the keratin and its materials. These macrofibrils are housed within disc‐shaped cells measuring 20–30 µm called keratinocytes, which adopt a layered structure (2 µm thick) at the microscale. These layers ultimately form a laminated structure containing tubules and continuous medullary cavities.

Considering these tubules, three different regions are observed: the intertubular area (the space between the walls of the tubules), the transition region (where the tubules intersect with the walls), and the tubule region [51]. Horse hooves contain a significant number of IFs embedded within the intertubular area, making up approximately 30% of this region. Such a densely packed structure provides exceptional mechanical resistance and cushioning properties.

The chemical characteristics and composition of horse hooves can obviously vary depending on nutrition, which may also impact vital functions and locomotion ability [35]. Dietary habits have been shown to influence the amino acid content of keratin, directly affecting the three‐dimensional structure of keratin, which in turn influences its properties and functions [31].

To the best of our knowledge, only a few articles have examined the amino acid composition of the protein fraction in horse hooves, highlighting the limited interest of the scientific community and the general public in this material as a protein‐fraction source. Glutamic acid, arginine, and serine were the major amino acids identified, together accounting for 31.2–33.8% of total amino acids, followed by leucine (8.07–9.21%), aspartic acid (6.55–7.90%), proline (4.56–8.40%), glycine (4.66–6.98%), and valine (5.21–6.40%). As previously mentioned, cysteine is crucial in keratin due to its sulfur content, and it was found in horse hooves at concentrations of 6.00–7.28%. Other amino acids found in lower amounts include alanine (4.19–5.99%), tyrosine (2.02–4.47%), as well as the essential amino acids (EAAs) threonine, isoleucine, lysine, phenylalanine, histidine, and methionine [52].

### 5.2 Mineral Content

The mineral composition of the horse hoof varies depending on factors such as breed, environment, and diet. These minerals play a crucial role in the keratinization process during hoof growth. Calcium (Ca) functions as an enzyme cofactor in keratinization, regulating cellular differentiation and the desquamation of epidermal keratinocytes. It also influences keratin's hardness based on the crystal structure formed and aids in the cross‐linking of sulfur bonds between proteins, promoting cell cohesion [30]. Copper (Cu) contributes to the physical properties of keratin by participating in the formation of disulfide bonds, which act as structural bridges within the keratinous protein matrix, which is verified both in horse hoof and in bovine claws [34]. Zinc (Zn) plays a key role in keratin synthesis and hoof formation, enhancing resistance to stress [34]. Due to this role of minerals in keratinization of hoof cells, mineral deficiencies can induce hoof structural changes, thus serving as hoof health and integrity indicator [53].

Mineral composition data from studies in the literature on horse hooves are compiled in Table 1: iron (Fe), sulfur (S), potassium (K), and calcium (Ca) are the major minerals, followed by sodium (Na), magnesium (Mg), manganese (Mn), phosphorus (P), aluminim (Al), and zinc (Zn), with smaller amounts of copper (Cu), nickel (Ni), strontium (Sr), lead (Pb), selenium (Se), and lithium (Li).

**TABLE 1:** Mineral composition of horse hooves compiled from the literature. Data are presented in ppm, except for values reported by Spörndly‐Nees et al. [42], which are expressed in g/kg (except for Cu, Mn, and Zn, expressed in mg/L).

	[42]	[40]^a^	[35]^a^	[41]^a^	[30]	[34]	[54]	[55]
**Al**	238 ± 99	324 ± 198	n.d.	n.d.	n.d.	n.d.	n.d.	n.d.
**Ca**	1467 ± 121	1138 ± 280	1.10 ± 0.14^b^	1178 ± 320	426 ± 486	631 ± 418	n.d.	179 ± 137
**Cu**	5.7 ± 0.6	4.3 ± 1.4	25 ± 6	5.5 ± 0.9	5.5 ± 1.2	1.8 ± 1	0.05 ± 0.00	4 ± 2
**Fe**	2509 ± 389	942 ± 587	n.d.	1268 ± 250	376 ± 373	114 ± 145	n.d.	291 ± 256
**K**	2201 ± 175	1822 ± 431	1.8 ± 0.3^b^	1020 ± 88	1179 ± 1119	3416 ± 3270	n.d.	461 ± 425
**Li**	0.7 ± 0.0	0.46 ± 0.10	n.d.	n.d.	n.d.	n.d.	0.01 ± 0.00	n.d.
**Mg**	373 ± 33	278 ± 58	0.3 ± 0.0^b^	455 ± 66	1772 ± 760	181 ± 98	n.d.	960 ± 632
**Mn**	326 ± 34	118 ± 68	23 ± 7	64 ± 11	n.d.	n.d.	n.d.	n.d.
**Na**	379 ± 52	358 ± 79	0.5 ± 0.0^b^	n.d.	250 ± 135	2242 ± 1102	n.d.	410 ± 143
**Ni**	5.5 ± 0.6	2.3 ± 1.0	n.d.	n.d.	n.d.	n.d.	n.d.	n.d.
**P**	263 ± 28	208 ± 43	0.3 ± 0.0^b^	315 ± 14	n.d.	n.d.	n.d.	n.d.
**Pb**	2.7 ± 0.3	1.8 ± 0.5	n.d.	n.d.	n.d.	n.d.	0.00 ± 0.00	n.d.
**Se**	1.2 ± 0.2	0.4 ± 0.3	n.d.	n.d.	n.d.	30 ± 34	n.d.	n.d.
**Sr**	5.6 ± 0.5	3.7 ± 1.0	n.d.	n.d.	n.d.	n.d.	2.1 ± 0.4	n.d.
**Zn**	132 ± 6	117 ± 17	168 ± 7	134 ± 13	146 ± 42	79 ± 55	1.08 ± 0.07	164 ± 37
**S**	n.d.	n.d.	22.6 ± 0.4^b^	18 968 ± 636	n.d.	n.d.	n.d.	n.d.

Abbreviation: n.d., not determined.

a Estimated means and standard deviations derived from data reported in the cited studies for various evaluated parameters.

b Expressed in g/kg.

As noted, the mineral composition of horse hooves is influenced by multiple factors. For instance, hoof color does not affect mineral content [41, 54], whereas diet (*p* < 0.05) [41, 55], geographic region (*p* < 0.05), and hoof sampling area significantly impact mineral composition [54]. Seasonal variations also affect mineral content, with significant differences in Ca, Mg, and Zn levels observed based on the time of the year (*p* < 0.01) [41]. Similarly, K, Mg, and Mn levels were higher in hooves from live horses compared to culled horses (*p* < 0.01) [35]. Age also plays a role, with significant differences (*p* < 0.05) detected in all analyzed minerals except Mg [34]. For example, Zn was found in higher concentrations in younger horses (under 3 years old) compared to those aged 3–5 years, whereas Na levels were lower (*p* < 0.05) [16]. Additionally, hooves from female horses exhibited significantly higher Zn levels (*p* < 0.05), while working or racing horses had higher Mg levels than reproductive horses (*p* < 0.05) [16]. Regarding racing conditions, hooves from horses that raced in the previous year contained higher K, Mg, and Mn levels compared to non‐racing horses (*p *≤ 0.05). Moreover, horses that raced occasionally without shoes had higher Cu levels (*p* = 0.02) than those racing barefoot frequently [34]. Although no significant differences were observed in total ash content based on breed (*p *≥ 0.01), significant variations in mineral composition were reported (*p* < 0.05) [26, 34, 40, 42].

Another factor to consider when assessing the mineral composition of horse hooves is the environment. Weather conditions can also impact, as high rainfall during the sampling period can create a highly acidic environment, leading to increased Fe solubilization and, consequently, greater absorption in the horse hoof [56]. Furthermore, proximity to industrial areas may be associated with elevated Pb levels in horse hooves, as long‐term exposure, even at low levels, can contribute to the accumulation of Pb in tissues over time [55].

## 6 Horse Hooves Properties

Horse hoof wall properties have been recently characterized either for speculative interest or as a starting point of bioinspired materials development. Literature investigations focus on mechanical properties and water sorption and desorption behavior: a general summary of the findings related to both characteristics is provided here.

### 6.1 Water Sorption and Desorption

The properties of keratin‐based materials are strongly influenced by the degree of hydration: both excessively low and high humidity have a detrimental effect on hoof wall tensile strength [49]. For this reason, non‐mechanical functions such as proper hydration maintenance and insulation are also of paramount importance for this material and have been deeply investigated, as described in the dedicated section.

The keratin‐based structure of the hoof wall is subject to varying hydration conditions, which significantly influence its mechanical properties. In vivo, the hoof wall experiences two distinct hydration gradients: a horizontal gradient, where the outer surfaces are drier than the interior, and a vertical gradient, where hydration decreases from the dermis region toward the ground [46].

The hoof wall's ability to retain water and the resulting changes in mechanical properties depend on its unique molecular structure. Hydrogen bonds play a crucial role in stabilizing the *α*‐helix structure of keratin molecules and partially contribute to cross‐linking adjacent polymer chains [48]. In fully dehydrated samples, extensive hydrogen bonding increases secondary cross‐linking, reducing matrix mobility. Conversely, in fully hydrated samples, water infiltration breaks hydrogen bonds, elongating the distances between cross‐link positions and allowing greater molecular movement [46].

Although exact in vivo hydration levels remain unknown, measurements from freshly harvested samples suggest an average water content between 17% and 24% [57]. Due to the strong dependence of mechanical properties on hydration, researchers have investigated the hoof wall's water sorption and desorption behavior, as well as the relationship between external humidity (stimulus) and water content (response), as summarized in Table 2. However, many studies lack details regarding experimental procedures, particularly equilibration conditions, making direct data comparisons difficult.

**TABLE 2:** Hydration levels after equilibration in different conditions of relative humidity (RH) compiled from the literature. Data are presented in % water.

Equilibrating conditions	% water	Reference
100% RH	40.2 ± 2.7	[46]
75% RH	18.2 ± 0.3	[46]
53% RH	11.7 ± 2.7	[46]
0% RH (2–3 weeks)	5.5 ± 1.8	[46]
*Fully hydrated samples* 100% RH (4 days)	35 ± 4	[57]
*Ambient samples* 27–40% RH	18 ± 3	[57]
*Fully hydrated samples* 100% RH (3 days)	~40	[8]
*Fresh samples*	~30.2	[8]
*Ambient dry*	~8.8	[8]
*Fully hydrated samples* 100% RH (24 h)	n.d.	[50]
*Ambient samples* 75% RH	~18.2	[50]
*Dry samples*	n.d.	[50]

Despite methodological differences and missing details, some general conclusions can be drawn. The hoof wall's maximum water absorption capacity is approximately 40%. Even under extreme drying conditions, complete water removal is not possible, suggesting the presence of tightly bound water molecules within the polymeric matrix [58], which influence the mechanical properties of keratin [59]. These findings are essential for comparing mechanical properties under different conditions and approximating the actual in vivo state.

### 6.2 Mechanical Properties

As described in the previous section, the hoof wall has a composite structure where crystalline IFs are embedded in an amorphous matrix composed mainly of keratin‐associated proteins with high sulfur content [8]. Several factors affect the mechanical properties of the hoof wall, including location, hydration level, IF dimensions, proportion, and orientation. Studies on hoof morphology and in vivo mechanical behavior have explored the impact of these factors on hoof performance [8, 39, 49, 57, 60].

Based on in vivo performance, hoof wall characterization has primarily focused on tensile, compressive, and relaxation properties, particularly their dependence on hydration and orientation. Hydration levels strongly influence these properties: all studies agree on a ductile‐to‐brittle transition with decreasing water content. The material behaves as an elastomer at higher hydration levels, becoming a rigid, glassy polymer when dehydrated [22]. Orientation has a weaker influence on macroscale samples, suggesting that anisotropy due to IF alignment is negligible [57].

Regarding tensile properties, Bertram et al. reported that the hoof wall's Young's modulus varies between 410 MPa and 14.6 GPa, depending on hydration levels [46]. Chen et al. later confirmed this range, finding values between 8.1 and 5.49 GPa across different hydration levels and structural regions (tubular or amorphous matrix) [49]. Rueda‐Carrillo et al. reported tensile strengths between 14 and 22 MPa [34]. More detailed studies have examined the dynamic response to tensile loading across strain rates from 10^−4^ to 100 s^−1^, consistently showing improved elasticity at higher frequencies [39, 50, 57].

Moving to compressive properties, various methods have been used to assess the fracture resistance of keratinous materials, yielding diverse metrics such as work of fracture [46], critical flaw size [46], loss tangent [57], storage modulus [57], and load–displacement plot [39, 61]. Due to differences in characterization protocols, direct numerical comparisons are difficult. However, in optimal hydration conditions, hoof wall toughness is approximately one order of magnitude higher than that of bone and twice that of wood [46]. Furthermore, hydration‐related differences can cause a two‐ to fourfold decrease in compressive strength [57].

Relaxation tests, though less commonly performed, provide insight into the material's ability to dissipate strain over time. Bonney et al. observed linear relaxation for small strains (2.5–5%), where the relaxation modulus was independent of the applied strain [57]. At larger strains, hydration level influences relaxation behavior, correlating with elastic modulus variations. As with compressive properties, optimal hydration (~20% water content) yields the best relaxation performance, with more than 75% of initial stress dissipating within 10 min [39]. In contrast, dry samples exhibit minimal viscoelastic relaxation and behave more like rigid plastics, while hydrated samples have significantly lower Young's modulus and higher absolute stress levels under the same strain [39].

## 7 Keratin from Horse Hooves: Toward Extraction and Applications

In this section, a brief overview of keratin extraction methods is provided, followed by a summary of proposed procedures and results on similar wastes, mainly hooves and horns, and possible applications for the extracted keratin or for raw hooves powder, as discussed for other keratinous wastes.

### 7.1 Overview of Keratin Extraction Methods

Keratin extraction methods include chemical (acid/alkaline hydrolysis, oxidation, reduction, and ionic liquids) and biological approaches:

• Acid and alkaline hydrolysis break sulfate and carboxyl groups at high temperatures, dissolving keratin and converting cysteine into cystic acid. Temperature and extraction time must be controlled to minimize peptide degradation [27]. Acid hydrolysis, consisting of strong acids such as HCl and H_2_SO_4_, is an efficient extraction method, but it reduces the nutritional value of the obtained peptide fraction and leads to the degradation of amino acids like tryptophan [2]. More prolongated extraction increases yield but produces lower molecular weight compounds. Alkaline hydrolysis, using hydroxyl compounds such as Ca(OH)_2_, KOH, and NaOH, preserves amino acids but weakens keratin's structure due to decarboxylation, desulfuration, and deamination. In general terms, efficiency depends on pH, temperature, and time of the extraction, while combining both methods improves efficiency but generates significant acid/base waste, impacting equipment and the environment [1].
• Oxidation uses oxidizing agents such as performic acid, paracetic acid, hydrogen peroxide, sodium percarbonate, mild ammonia, and HCl to break the disulfide bonds in keratin, converting cysteine into cysteic acids and forming a water‐soluble, noncovalent structure called keratoses. Higher reaction times and oxidant concentrations improve extraction yields but can degrade amino acids such as tryptophan, cysteine, serine, and threonine [27, 62].
• Reduction method uses agents such as thioglycolic acid, thiosulfates, 2‐mercaptoethanol [63], and L‐cysteine to break disulfide bonds (R‐S–S‐R) of cysteine chains, forming free thiols (R‐SH) of cystine. Protein denaturants such as urea enhance disulfide bonds’ accessibility to the reducing agents by breaking hydrogen bonds and hydrophobic interactions [3]. However, cysteine products can reoxidize, requiring surfactants such as sodium dodecyl sulfate (SDS), iodoacetic acid, or ethylenediaminetetraacetic acid (EDTA) to prevent reversal by forming micelle structures [27]. While widely used for *α*‐ and *β*‐keratin extraction, this method has drawbacks, including high cost, toxicity, nonrecyclability, and environmental impact [1, 27].
• Ionic liquids, existing only in their ionic form, offer higher solvation capacity than traditional solvents. Their strong polarity disrupts hydrogen bonds in keratin, aiding dissolution. Cosolvents such as urea or SDS help break disulfide bonds, boosting extraction yield [64].
• Biological keratin extraction relies on enzymes from microorganisms such as bacteria, actinomycetes, and fungi, as well as extracted and synthesized enzymes. While common proteases cannot break down the keratin's rigid structure, keratinases are the most effective [65]. Their efficiency depends on substrate concentration, enzyme type and concentration, and extraction time and temperature. Since disulfide bonds hinder enzyme access, pretreatment (e.g., reducing agents, autoclaving, or alkaline treatment) is often required [27]. Compared to chemical methods, keratinase extraction is eco‐friendly [27], requiring low energy and mild conditions while minimizing amino acid loss. However, it produces lower molecular weight peptides, limiting applications, and enzyme costs remain high [1].

Other novel keratin extraction methods include microwave and ultrasound‐assisted extraction, steam explosion, pulsed electric fields, and superheated and high‐pressure processes [1, 2, 3].

### 7.2 Keratin Extraction from Hooves and Horns

Extraction methods for keratin from hard animal tissues typically involve similar chemical or physical treatments aimed at disrupting the highly cross‐linked protein structure. Given the current absence of specific peer‐reviewed data detailing keratin extraction methods applied directly to horse‐hoof trimming waste, we present data for cattle hooves and horns in Table 3, as the most relevant structural and chemical proxy.

**TABLE 3:** Extraction of keratin from cattle hoof and horn wastes using different extraction methods.

Source	Pretreatment	Extraction method	Post‐extraction treatment	Quantification techniques	Extraction yield	Ref.
Bovine hooves	Grinding, washing (water, x3), oven‐drying, hexane‐dichloromethane (1:1)‐defatting (2 days)	7M urea, 6 g sodium dodecyl sulfate, 15 mL 2‐mercaptoethanol (60°C, 12 h, pH 7)	Centrifugation (6000 rpm, 15 min), dialysis (5–6 days), freeze‐drying	Carbon, hydrogen, nitrogen and sulphur analysis	44% E.Y.	A [59]
Bovine hooves and horns	Grinding, sieving, autoclaving (5 g keratin meal in 50 mL Trizma buffer pH 8, 135°C, 30 min, 0.26 MPa)	5 g keratin meal (1:67‐1:17 enzyme/substrate ratio) in the enzyme solution (75–300 mg keratinase in 100 mL 0.05M Trizma buffer (pH 8)) (50°C, 5‐6 h). If autoclaving: 50 mL of Trizma buffer (pH 8) containing the enzyme (0.75–3.00 g/L)	Samples for analysis: ice‐water bath cooling, centrifugation	Bicinchoninic acid assay, 560 nm absorbance (soluble protein), Kjeldahl method (nitrogen content), amino acid analyzer (free amino acids)	59.3% soluble nitrogen, 5.54 g/L soluble proteins (opt.: 3.0 g/L enzyme), 6.5 g/L amino acids (opt.: 1.50 g/L enzyme)	B [66]
Cattle hooves	Washing (water), oven‐drying (100°C, 5 h), grinding, sieving (≤1 mm), defatting	Experimental design: time (60, 120, and 180 min), temperature (55, 65, and 75°C), NaOH (0.3, 0.65, and 1M) with 0.64 g of urea	Filtration, precipitation (1N HCl, pH 4), freeze‐drying	Kjeldahl method (purity of keratin), weight differences b/a drying (E.Y.)	85% E.Y. and 89.6% purity (opt.: 60 min, 55°C, 0.5M NaOH)	C [28]
“Horn‐hoof” fertilizer	Washing (sodium dodecyl sulfate, water, acetone), conditioning (20°C, 24 h, 65% humidity).	D. 5 mM Tris (pH 8.6 HCl), 0.14M dithiothreitol, 8M urea (4 h, nitrogen atmosphere) B. 8 M urea, 0.5M meta‐bisulfite, 0.05M SDS, pH 6.5 5N NaOH (65°C, 2 h, 1:20 solid/solvent ratio)	Filtration	Bradford assay	3% (A) and 10% (B) E.Y.	D and E [67]
Goat hoof	Washing (water), grinding	25 mM Tris (pH 8.5 with HCl), 5M urea, 2.6M thiourea, 12.5 mL thioglycolic acid), pH 7.7–9 with 5M NaOH (1:25 solid/solvent ratio, 50°C, 48 h)	Filtration, centrifugation (5000 rpm, 15 min), dialysis (room temperature, 3 days), rotator‐vacuum evaporation	n.r.	n.r.	F [68]

This choice is justified by the fact that both horse hooves and cattle hooves or horns are primarily composed of hard *α*‐keratin with a similar hierarchical, laminated, and highly cross‐linked structure. Although minor differences in composition among animal species, especially concerning cysteine content or lipid profile, are expected, due to varying factors such as diet and management, the fundamental chemical and physical principles required to solubilize this resilient *α*‐keratin, mainly reduction, oxidation, or alkaline/acid hydrolysis, are directly transferable across these hard keratinous tissues. Therefore, Table 3 serves to illustrate the established methodologies most likely to be applicable to horse‐hoof trimmings, providing a necessary reference for future research.

Veselá et al. explored the use of keratinases for extracting keratin from bovine horns and hooves, incorporating an autoclaving stage to facilitate enzyme access to peptide bonds, achieving extracts with up to 60% of soluble nitrogen [66]. The lowest extraction yields were reported when using reducing agents such as 2‐mercaptoethanol and dithiothreitol, yielding 44% [59] and 3% [67], respectively. In contrast, the highest yields were reported by Yilma et al., who used NaOH and urea as denaturants, achieving an 85% extraction yield with over 85% purity [28]. In all cases, after extracting keratin, precipitation was carried out by lowering the pH to an acidic level below keratin's isoelectric point. This was followed by filtration, centrifugation, or dialysis and then freeze‐drying to obtain pure powder keratin.

### 7.3 Current Applications of Keratin from Hooves

More specifically, keratin derived from hooves has been largely overlooked since only a few studies have explored its potential, suggesting applications in biomaterials for biomedical fields such as regenerative medicine [67, 68] and as a foliar fertilizer, as a source of proteins, peptides, and free amino acids [66]. However, the remarkable impact resistance and energy absorption properties of the horse hoof wall have served as bioinspiration for materials development. Some examples already exist, such as in [45]: the advantage of this approach in terms of recycling is that the knowledge of the mechanical performance of the whole structure also gives suggestions for the application of recycled (powder, shavings) as such into a “host” material, not aiming necessarily at the extraction of keratin.

Another example is described by Rice and Tan, who designed a 3D polylactic acid (PLA) layered material inspired by the lamellar structure of the horse hoof, enhancing its resistance to fracture propagation by directing cracks along the interlayer [69]. Wang et al. replicated the tubular structure of bovine hooves to create a 3D‐printed material with improved mechanical properties [70]. Lastly, Ma et al. developed structures inspired by the tubular architecture of the horse hoof wall, achieving superior crash impact protection and greater energy absorption compared to traditional materials [71].

### 7.4 Potential Applications of Raw Hoof Powder

Besides using horse hoof waste as raw material to be submitted to extraction protocols to obtain keratin, other direct applications of horse hoof powder should be explored, in parallel with similar studies on other keratin‐based sources. For example, the horse hoof waste, being a hard, rigid particulate *α*‐keratin source, could offer a superior advantage over soft fibrous keratin sources (wool, feathers) when utilized as a filler. The material's intrinsic high modulus and geometry, derived from its native cellular structure, could allow it to act as an effective structural reinforcing agent in thermoplastic matrices (e.g., PLA, polybutylene succinate, and polyvinyl chloride), suggesting similar or even higher performances than traditional keratinous wastes, such as bovine claw powder and wool keratin powder [72, 73, 74, 75, 76]. This specific rigidity facilitates the development of bio‐based structural composites requiring improved mechanical and thermal stability for demanding applications, such as in the automotive, construction, or specialized packaging sectors.

The keratin sourced from horse hoof trimmings benefits from being a cruelty‐free, highly traceable feedstock from a managed care stream, ensuring high quality and consistency, which is crucial for sensitive biomedical applications. The resulting soluble keratin can be used to fabricate high‐quality hydrogels, films, and porous scaffolds for applications such as wound dressings, drug delivery, or tissue engineering. The purity and consistency of this source material minimize the risk of contaminants often associated with slaughterhouse by‐products, while the complex, laminated structure of the hoof continues to inspire the design of new hierarchical, fracture‐resistant materials. It goes without saying that safety issues must be strongly discussed and granted before aiming at a similar application considering the intrinsically “dirty” nature of waste materials.

Lastly, the chemical structure of keratin is rich in reactive functional groups, especially cysteine residues (thiol groups, —SH). Due to this chemical affinity, appropriately prepared hoof keratin powder, which is potentially generated in high volumes, could serve as a highly effective and low‐cost biosorbent, as similarly proposed for sheep hooves powder [72]. These materials might show significant potential for the selective removal of heavy metal ions and organic pollutants from wastewater, offering a biodegradable and high‐capacity alternative to conventional filters.

## 8 Toward Horse Hooves Upcycling

### 8.1 Projected Volume of Keratin Sourced from Equine Hoof Trimmings as a Feedstock

As there are no official data available in the literature regarding both keratin extraction from horse hoof samples and the volume of horse hooves produced, it is not possible to accurately estimate the potential amount of keratin that could be obtained from this feedstock or its impact on the keratin market. However, an initial estimation can be made based on the previously estimated yearly production (106 000 tons), the average moisture content between 17% and 24% [58] and an extraction yield comparable to the ones reported for cattle hooves (41% as average yield among the various protocols summarized in Table 3). This preliminary estimation could result in annual production of between 33 and 36 thousand tons of keratin, which could significantly help meet the projected increase in demand for proteins, biopolymers, and bioplastics in the coming years, while simultaneously reducing environmental impact by utilizing this alternative bio‐based protein source. Therefore, further research is needed, not only on the keratin extraction process from horse hooves but also on the scaling opportunities to implement this process at an industrial level.

### 8.2 Sustainability Assessment Metrics for Keratin Extractions

Keratin extraction from chicken feathers and sheep wool has been widely studied, but few studies have explored its recovery from cattle hooves, with no LCA evaluations conducted. Additionally, no research has been published so far on keratin extraction from horse hooves, highlighting a research gap in both utilizing horse hooves as a natural keratin source and assessing the sustainability of these processes. Adopting eco‐friendly extraction technologies is crucial to minimizing waste and environmental impact, fostering a circular bio‐based economy in line with green chemistry principles.

To first address these limitations, we adopted the Green Extraction Tree (GET) methodology [77]. Unlike scoring systems developed for analytical chemistry (such as AGREE or Green Analytical Procedure Index (GAPI)), the GET is specifically designed to evaluate the environmental impact of green extraction processes from natural products [77]. The GET framework assesses the process based on five key domains (raw material, solvent, energy, process, and final product), with each being weighed in relation to the green extraction principles. The application of this tool ensures that our conclusions regarding the “greenness” of hard *α*‐keratin extraction protocols are based on a validated methodology pertinent to biopolymer process evaluation. The analysis particularly focused on parameters related to solvent hazard, energy demands (temperature/time), and nonrecycled waste generation.

**FIGURE 1:**
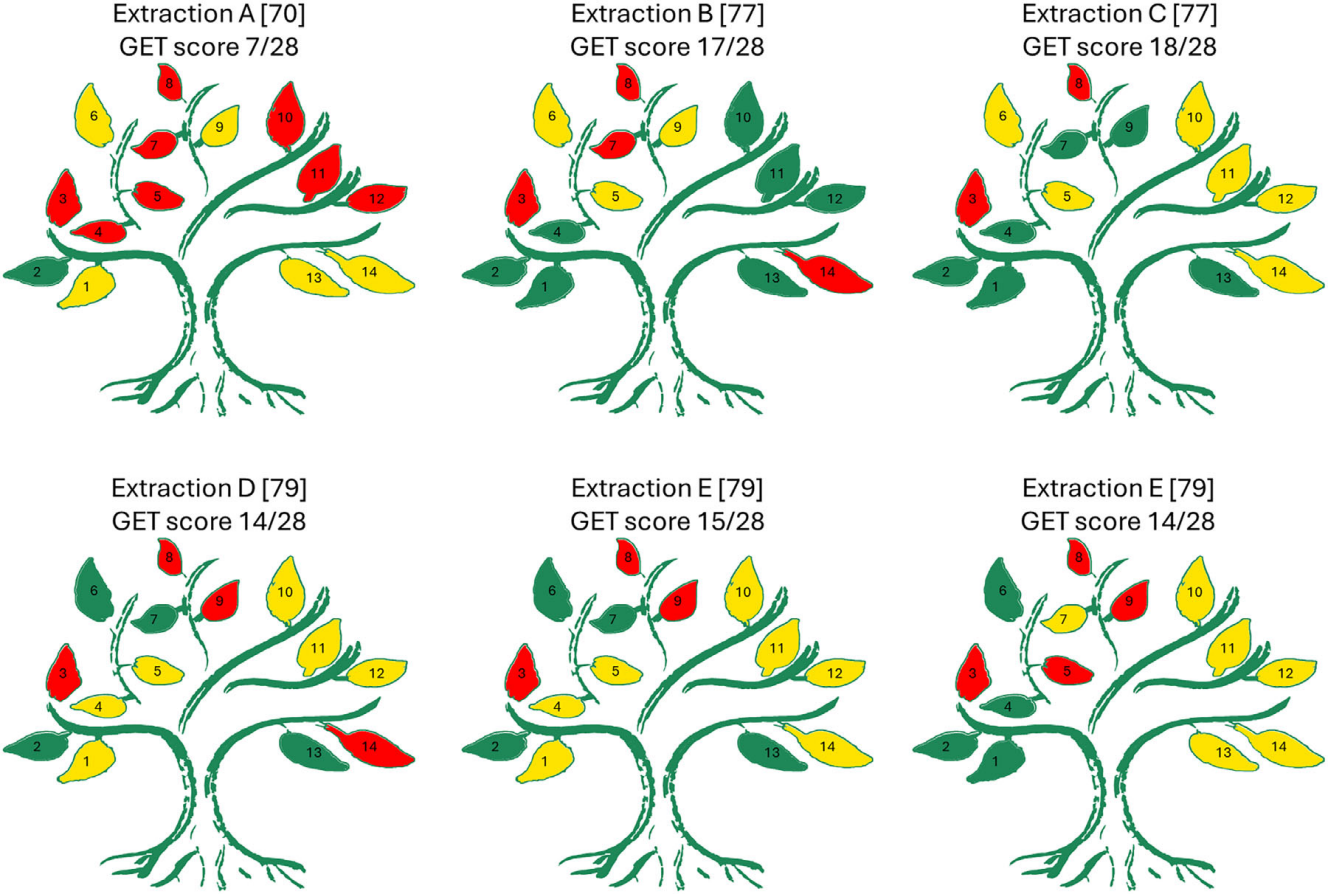
Greenness Score (GS) estimated using the GET methodology [77] for keratin extraction processes from cattle hooves listed in Table 3: (A) bovine hoof [59], (B) bovine hooves and horns [66], (C) cattle hooves [28], (D) “horn‐hoof” fertilizer—extraction A, and (E) extraction B [67], (F) goat hoof [68].

Figure 1 presents the estimated GS for keratin extraction from cattle hooves (Table 3) according to GET methodology, with letters A‐F referring to the same order in Table 3 rows, while a brief discussion of the assumptions applied is here summarized.

• Criterion 1—Promote the Use of Renewable Materials: all the extractions use cattle hooves as sustainable raw materials, but solvent‐free protocols (B, C, and F) are preferred over solvent‐based procedures with an assigned score of 2 and 1, respectively.
• Criterion 2—Ensure Sample Stability and Simplify Sample Storage: hooves represent a highly stable starting material that does not degrade or decay under normal storage conditions, thus the highest score is applied to all the protocols.
• Criterion 3—Minimize Sample Amounts: all the protocols score at the minimum since a mass far above 1 g is always employed, most frequently 5 g (B, D, E, and F) but also 10 g (A).
• Criterion 4—Use Safer Solvents and Reagents: the summarized protocols significantly differ according to this criterion since A uses only petrochemical solvents, scoring 0, while D and E exploit both water and solvents, scoring 1, and the remaining protocols only include aqueous solutions, scoring 2.
• Criterion 5—Minimize Solvent and Reagent Amounts: protocols A and F require high solvents volumes, around 250 mL or more, while a more efficient process is guaranteed for the other procedures.
• Criterion 6—Minimize Solvent and Reagent Amounts: the protocols are divided into two groups according to this criterion. The first group, including procedures A, B, and C, scored 1 for the presence of defatting or autoclaving, while the remaining protocols score 2 since include only water‐based washing.
• Criterion 7—Minimize Energy Consumption: the worst score is assigned to procedures A and B, which include high energy‐consuming steps (Soxhlet apparatus and autoclaving), protocol C score 1 thanks to the usage of mild temperatures (50°C) for a long time (48 h), while the remaining extraction are preferable for the joint application of mild temperatures and short durations.
• Criterion 8—Maximize Sample Throughput: all the extractions score 0 for the long time required and, consequently, extremely low efficiency in terms of sample throughput.
• Criterion 9—Maximize the Extraction Efficiency of Target Compounds: the metric for this criterion is substantially based on the comparison with existing methods but, being in our case, the only existing methods under investigation in this comparison, we opted for assigning the worst score to the less efficient extractions (D, E, and F) and increasing scores for intermediate (1 assigned to A and B) and high yield (C).
• Criterion 10 —Minimize By‐product and Waste Generation: the best score is assigned to protocol B, which implies only water‐based solutions containing enzymes, while the worst one is given to protocol A for the large disposal of organic solvent; an intermediate score should be assigned to protocols C, D, E, and F, which produce aqueous solutions containing caustic reagents or urea.
• Criterion 11 and 12—Reduce the Risk of Health Hazards and Operational Safety Risks: also in these cases, the worst score is assigned to A for the use of organic solvents, the intermediate value to C, D, E, and F for the inclusion of NaOH and urea and the best one to B, which does not include any toxic substance.
• Criterion 13—Choose Greener Analytical Detection Techniques: studies estimating keratin yield through UV–vis spectrophotometry, weight differences, or elemental composition performed well in this criterion (B, C, D, and E), while the other methodologies are less preferable.
• Criterion 14—Ensure the Industrial Production Prospects: the score assignation for this criterion is quite critical since none of these methodologies is actually used in an industrial environment, but, considering the different complexity of extraction protocols, procedures A, C, E, and F seem to be more promising than the others.

Hence, although first attempts to extract keratin from various cattle hoof sources have shown low sustainability due to the use of hazardous solvents and reagents, optimizing the process with safer reagents and milder conditions enhanced its green profile. Moreover, enzyme‐assisted extraction, a greener extraction technique, has proven to be effective on keratin recovery from bovine hooves and horns, highlighting a research gap and an opportunity to explore other eco‐friendly alternatives, such as high‐pressure processes, microwave, and ultrasound‐assisted extraction, to valorize this bio‐based keratin waste. While a full life cycle assessment (LCA) remains the gold standard for process sustainability evaluation, its application can be cumbersome and possibly even unreliable, when few information is available, either on the starting material or on the protocols, as in this case. Nevertheless, the GAPI approach provides a necessary and methodologically sound comparative assessment of existing literature protocols, thereby guiding future dedicated experimental studies.

## 9 Conclusions and Future Challenges

Horse hooves constitute a renewable ecological by‐product with remarkable mechanical properties, generated in significant amounts. Despite being a rich source of proteins, composed almost entirely of *α*‐keratin, they remain underutilized due to limited research on their chemical composition and properties. This review highlights the need for further studies to explore horse hooves as a novel keratin source, aiming to unlock their potential as a sustainable protein alternative for a sustainable animal production system, being not only a source of supply totally cruelty‐free, but obtained by processes related to animal welfare. While some studies have examined their morphology, function, and mechanical properties, emphasizing their strength and rigidity, there is a lack of standardized testing criteria, making it difficult to compare findings across different studies. Furthermore, the scientific community has paid little attention to the valorization of these residues for high‐value protein extraction. As a readily available, low‐cost, and nontoxic protein source with excellent biodegradability, horse hooves present a promising yet largely overlooked research opportunity in material development.

## Author Contributions

**Lisa Rita Magnaghi,** **Raffaela Biesuz,** and **Esther Trigueros**: conceptualization. **Lisa Rita** **Magnaghi** and **Sara Mattiello:** methodology. **Lisa Rita Magnaghi**, **Esther** **Trigueros,** and **Sara Mattiello:** investigation. **Lisa Rita** **Magnaghi**, **Esther Trigueros,** and **Sara Mattiello**: original draft preparation. **Carlo Santulli** and **Sara Mattiello:** writing – review and editing. **Raffaela Biesuz** and **Carlo Santulli:** supervision.

## Funding

The authors has nothing to report.

## Conflicts of Interest

The authors declare no conflicts of interest.

## Data Availability

No new data were created.
